# Alignment-free microbial phylogenomics under scenarios of sequence divergence, genome rearrangement and lateral genetic transfer

**DOI:** 10.1038/srep28970

**Published:** 2016-07-01

**Authors:** Guillaume Bernard, Cheong Xin Chan, Mark A. Ragan

**Affiliations:** 1 Institute for Molecular Bioscience, and ARC Centre of Excellence in Bioinformatics, The University of Queensland, Brisbane, QLD 4072, Australia

## Abstract

Alignment-free (AF) approaches have recently been highlighted as alternatives to methods based on multiple sequence alignment in phylogenetic inference. However, the sensitivity of AF methods to genome-scale evolutionary scenarios is little known. Here, using simulated microbial genome data we systematically assess the sensitivity of nine AF methods to three important evolutionary scenarios: sequence divergence, lateral genetic transfer (LGT) and genome rearrangement. Among these, AF methods are most sensitive to the extent of sequence divergence, less sensitive to low and moderate frequencies of LGT, and most robust against genome rearrangement. We describe the application of AF methods to three well-studied empirical genome datasets, and introduce a new application of the jackknife to assess node support. Our results demonstrate that AF phylogenomics is computationally scalable to multi-genome data and can generate biologically meaningful phylogenies and insights into microbial evolution.

Phylogenomics is the study of evolutionary relationships via comparative analysis of genome-scale data, intersecting the fields of evolution and genomics. Phylogenomic methods are used to assess diverse biological hypotheses, e.g. the emergence and spread of antibiotic resistance among bacterial pathogens^1^, or the tree of life^2^. Evolutionary relationships are inferred based on sequence homology in a comparative gene-by-gene^3^, concatenated multi-gene^4^ or whole-genome approach^5^. Classical phylogenomic approaches consist of three main steps: clustering of homologous sequences, multiple sequence alignment (MSA) of each cluster, and inference of a phylogenetic tree based on the alignment, e.g. using maximum likelihood (ML) or Bayesian methods. MSA is a crucial step in these approaches. In carrying out MSA we implicitly assume that by inserting gaps and sliding blocks of sequence relative to each other, we can generate a position-wise hypothesis of homology across the entire length of the sequences. In reality, this may be unrealistic because genes and genomes are subject to recombination, inversion, rearrangement and lateral genetic transfer^6^.

These processes are intensified in most microbial genomes, where LGT, inversions and rearrangement are rampant^5^,^7^,^8^. These events violate models of nucleotide substitution across sequence positions and lineages, commonly specified in MSA-based approaches, and thus bias subsequent phylogenetic inference^9^,^10^. In addition, increasing numbers of microbial genomes are now available both from a broad taxonomic breadth^11^,^12^ and within individual taxa^13^. MSA does not scale well with genome-scale data, such that classical approaches will soon be impractical for large-scale comparative genome analysis.

Alternatively, *alignment-free* (AF) methods, based on comparison of sub-sequences of defined length (known variously as *k*-mers or *n*-grams) instead of full-length sequences, can be used^14^,^15^,^16^. AF methods utilise subsequences (*k*-mers) as the basis for calculating an overall pairwise distance between one sequence and another. These methods (Fig. 1) fall into two broad categories^15^: those based on the number or frequency of *k*-mers shared between two sequences, and those based on the length of matching *k*-mers. Furthermore the *k-*mers may or may not be required to match exactly. In general, match-length methods perform well in the comparison of highly similar sequences, due to the large proportion of exact matches. Algorithmically, most AF methods show linear-time complexity, but word-count methods are usually faster and, depending on the implementation, can require less memory than match-length methods.

Recent studies demonstrate the potential of AF approaches in the accurate inference of phylogenetic relationships^15^,^17^, with several methods more robust than MSA-based approaches against gene-level evolutionary scenarios including among-site rate heterogeneity, compositional biases, genetic recombination and insertions/deletions. The AF methods assessed were more sensitive to recent sequence divergence and sequence truncation^17^. Across diverse empirical gene-family datasets, AF approaches perform well for sequences sharing low divergence, at greater computation speed. However, the sensitivity of AF approaches to evolutionary scenarios at whole-genome level including genome rearrangements and LGT, and their scalability to multi-genome data, remain to be systematically investigated.

Classical phylogenetic methods employ heuristics, and iterative sampling strategies have been adopted to assess node support, among which the best-known are the non-parametric bootstrap^18^ in ML and the posterior probability of tree bipartitions in Bayesian inference^19^,^20^. In comparison, AF approaches provide exact solutions with no iterative processes, with the consequence that phylogenetic trees generated using AF approaches lack estimates of node support. Bootstrap and subsampling techniques have been proposed in recent studies^21^,^22^,^23^, but most studies of AF approaches focused only on topologies, with no or little emphasis on node support.

Here, using both simulated and empirical data we systematically assess the sensitivity of nine existing AF methods to genome-scale evolutionary scenarios involving sequence divergence, LGT and rearrangement. We introduce a new application of the jackknife^24^ technique to provide node-support values to trees inferred by AF approaches, and demonstrate the scalability and potential of AF approaches in inferring phylogenetic trees quickly and accurately from genome-scale data.

## Results

We investigated nine AF phylogenetic methods: 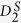 25, *co-phylog*^26^, *cvt*^27^, *ffp*^28^,^29^,^30^ and *spaced*^31^,^32^ based on word count, and *acs*^33^, *gram*^34^, *kmacs*^31^,^35^ and *kr*^36^ based on match length (see Supplementary Information). The distance matrix generated by each of these methods was used to infer phylogenetic relationships using neighbour-joining. As a measure of accuracy we used the Robinson-Foulds distance^37^, which evaluates the topological congruence between an inferred tree and a reference tree. We designate *RF* as the normalised Robinson-Foulds distance (see Methods): *RF* = 0 indicates that the test-tree topology is completely congruent with that of the reference, while *RF* = 1 indicates that the two trees have no bipartitions in common.

### Simulated data

Using simulated data, we independently assessed the sensitivity of each AF approach to evolutionary scenarios of genome divergence, LGT and genome rearrangement. Because the underlying tree on which each dataset has been simulated is known, we used that as the reference. Here we report, for each scenario, results generated with each AF approach using the parameter setting that yields the smallest mean *RF* globally across all rates; these settings are optimal in this context. The parameters of interest are *k* (*k*-mer length) for *cvt*, 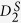 and *ffp; K* (half-context length) for *co-phylog*; *n* (number of patterns) for *spaced*; and *mm* (number of mismatches) for *kmacs*. We used default parameters for *acs*, *gram* and *kr*.

#### Genome divergence

**Figure 2:**
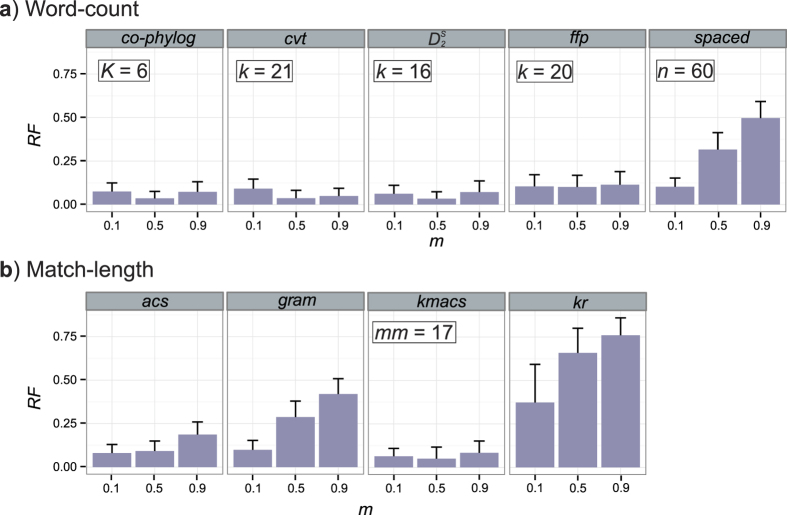
Accuracy of AF methods based on *m*. *RF* distances are shown for (**a**) word-count methods and (**b**) match-length methods at *m* = 0.1, 0.5 and 0.9. Error bars indicate standard deviation from the mean across 50 replicates.

We simulated sets of genomes (size *N* from 25 to 35) using a birth-death model across different levels of genome divergence as determined by the mutation rate *m* *=* 0.1, 0.5 and 0.9; *m* is the number of nucleotide substitutions per iteration. At maximum rate *m* = 1, almost all nucleotides are substituted per iteration^38^. Figure 2 shows the mean *RF* for each approach (50 replicates) across these values of *m*. Optimal settings for each approach were determined based on a wide range of parameters (see Supplementary Information and Supplementary Figure S1 for details). For all the word-count methods except *spaced*, *RF* is minimum at *m* = 0.5 (Fig. 2a), i.e. at an intermediate level of sequence divergence. On the other hand, for the match-length methods (except *kmacs*) *RF* increases with greater sequence divergence (Fig. 2b), suggesting that their accuracy in inferring the correct genome phylogeny decreases as the sequences become more dissimilar from one another.

Interestingly, the pattern of *RF* values observed for *kmacs* (*RF* = 0.06, 0.05 and 0.08 at *m* = 0.1, 0.5 and 0.9) follows that of most word-count methods, while the pattern of *RF* values we find for *spaced* (*RF* = 0.10, 0.31 and 0.48 at *m* = 0.1, 0.5 and 0.9) increases with greater sequence divergence, as with most of the match-length methods.

#### Lateral genetic transfer

We simulated genome sets at different extents of LGT as determined by the mean number of LGT events per iteration *l* = 0, 5, 25, 125, 250, 500; *l* is the mean number of LGT events attempted in the set at each iteration (*i* = 5000) during the simulation. Thus at *l* = 5, the simulator attempted five LGT events per iteration across a genome set (see Methods). LGT events were simulated to occur at random, i.e. anywhere along a genome sequence and between any pair of genomes in a set.

**Figure 3:**
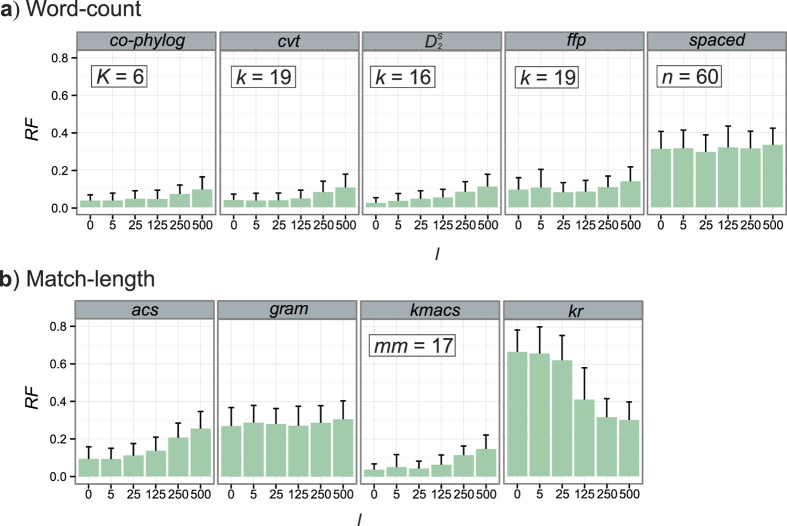
Accuracy of AF methods based on *l*. *RF* distances are shown for (**a**) word-count methods and (**b**) match-length methods at *l* = 0, 5, 25, 125, 250 and 500. Error bars indicate standard deviation from the mean across 50 replicates.

Figure 3 shows the mean *RF* for each AF method (50 replicates) across different values of *l*. Optimal settings for each method were determined by tuning a range of parameters (Supplementary Information and Supplementary Figure S2). In general, word-count methods (Fig. 3a) achieved lower *RF* values than did match-length methods (Fig. 3b) against the complication of LGT. Not surprisingly, for most AF methods the accuracy of inference falls off with increasing extent of LGT. Several methods were nonetheless able to reconstruct the reference tree with little incongruence at all but the two highest frequencies of LGT simulated, notably *co-phylog*, *cvt*, and *kmacs* (*RF* < 0.10 at *l* ≤ 125; Fig. 3).

The word-count method *spaced* (Fig. 3a) and the match-length methods *gram* and *kr* (Fig. 3b) were relatively inaccurate (*RF* > 0.2 across *l*) by this measure. Although *spaced* and *gram* are robust against variation of *l*, the inaccuracy of *kr* decreased with increasing *l* while remaining unacceptably high. As mean *RF* values approaching 1.0 indicate that many of the inferred topological features are scarcely distinguishable from those of a random tree^39^, it appears that our simulation scenario was too extreme for *kr*.

**Figure 4:**
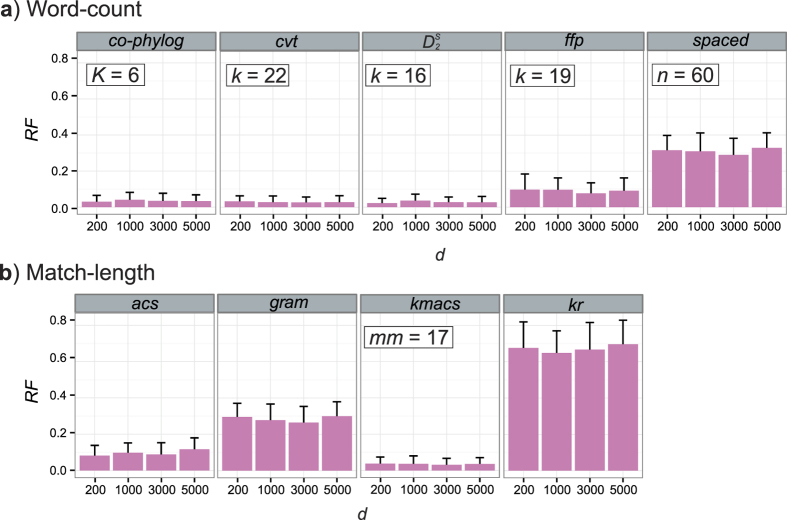
Accuracy of AF methods based on *d*. *RF* distances are shown for (**a**) word-count methods and (**b**) match-length methods at *d* = 200, 1000, 3000 and 5000. Error bars indicate standard deviation from the mean across 50 replicates.

We also varied the maximum evolutionary distance within which LGT can occur. At fixed *l* = 5 we simulated LGT while setting the divergence factor *d* = 200, 1000, 3000 or 5000, where *d* is the maximum number of iterations (generations) that can separate two genomes from their common ancestor for a proposed LGT event to be accepted. This simulates biological situations in which genetic material can be successfully transferred only among related organisms due to e.g. limited plasmid host range, or a threshold of local sequence identity below which homologous recombination is not successful. Figure 4 shows the mean *RF* for each AF method (50 replicates) across *d* at otherwise optimal parameter settings (Supplementary Information and Supplementary Figure S3). For both word-count (Fig. 4a) and match-length (Fig. 4b) methods, *RF* varies little with *d*. Thus the effectiveness of these AF methods to reconstruct the reference tree in the presence of LGT is little affected by relatedness of the sequence donor.

#### Genome rearrangement

We simulated genome sets as above (see Methods for details) at different frequencies of genome rearrangement *r* = 0.00, 0.01, 0.10 or 1.00 (each in 50 replicates). A rearrangement event was simulated as an inverted translocation occurring at any position of a genome. The latter three frequencies correspond to rearrangement of approximately 0.20%, 2.0% and 20% of each genome sequence (by length). As above, we report results generated using the parameter settings optimal for each AF method (see Supplementary Information and Supplementary Figures S4 and S5).

**Figure 5:**
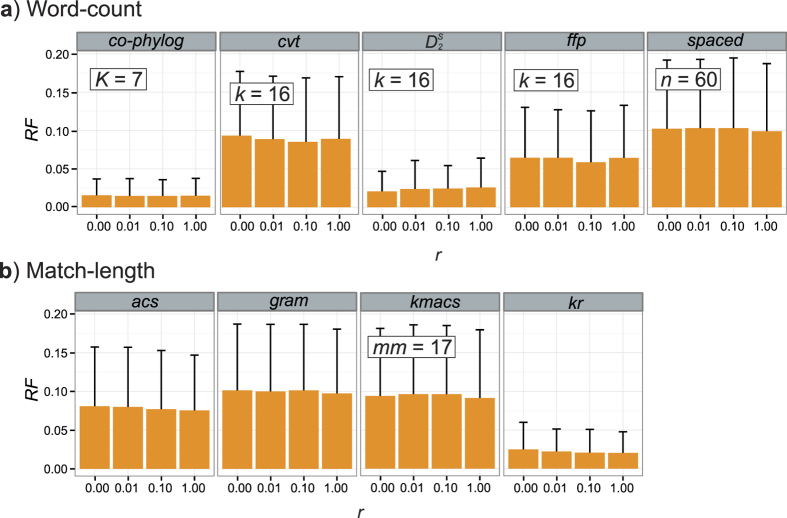
Accuracy of AF method based on *r*. *RF* distances are shown for (**a**) word-count methods and (**b**) match-length methods at *r* = 0.00, 0.01, 0.10 and 1.00. Error bars indicate standard deviation from the mean across 50 replicates.

Figure 5 shows the mean *RF* for each method across these values of *r*, at optimal parameters. Three of these AF methods, *co-phylog,* 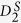 and *kr*, were affected by these simulated rearrangements much less (*RF* < 0.03 across *r*) than were the others (*RF* > 0.05). No trend with *r* was apparent. These results extend an earlier study^17^ in which AF methods were likewise found to be robust against genetic recombination at the level of individual genes.

### Empirical data

To assess the performance of the AF methods in application to empirical data, we used three sets of microbial genome sequences: (a) 143 bacterial and archaeal genomes^8^ previously used to infer the extent of LGT across divergent taxa, (b) 27 genomes of *Escherichia coli* and *Shigella*^3^ used to infer LGT among more-closely related taxa, and (c) eight *Yersinia* genomes that are too similar in sequence for classical phylogenetic inference, but share patterns of genome rearrangement^5^ (see Methods and Supplementary Information for details). As references we used the MRP supertree^40^ summarising the well-resolved subtrees inferred by a standard MSA-based approach from single-copy protein sets for the former two studies^3^,^8^, and the consensus phylogenetic network based on genomic inversion events inferred from a whole-genome alignment for the *Yersinia* dataset^5^. For each AF tree we computed the jackknife (JK) support for each node across 100 pseudo-replicates (see Methods).

#### 143 bacterial and archaeal genomes

**Figure 6:**
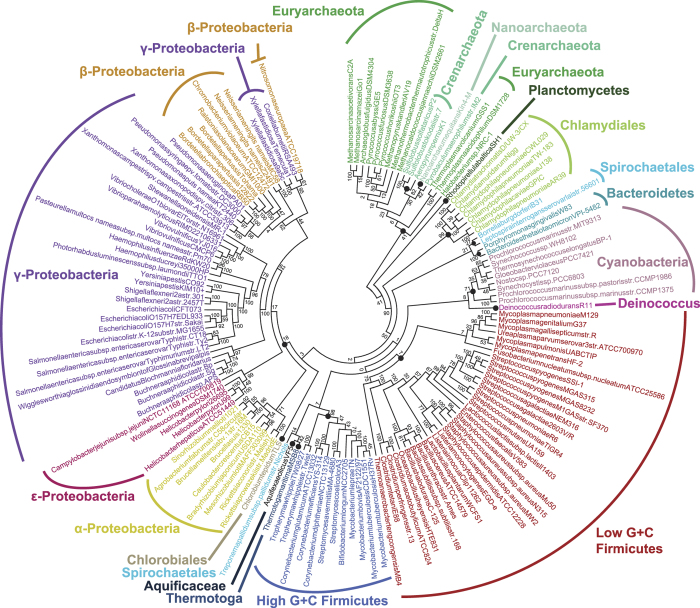
AF phylogeny of 143 prokaryote genomes. Phylogenetic tree of 143 prokaryote genomes using 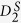 at *k* = 24, supported by JK values. The 15 phylum-level backbone nodes of Beiko *et al*.^8^ are marked with solid circles.

Figure 6 shows the 143-taxon tree inferred using an AF approach (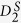 at *k* = 24), with the JK support for each node. Among the trees we inferred using other AF methods and parameter settings (see Supplementary Information and Supplementary Figure S6), this tree shows the greatest topological similarity (*RF* = 0.42) to the reference supertree^8^. Although 42% of bipartitions are incongruent with the reference, we recover 13 of the 15 phylum-level “backbone” nodes identified by Beiko *et al*.^8^ (Fig. 6), some with very strong JK support (e.g. Bacteroidetes 100%, Chlamydiales 99%, high G + C Firmicutes 98%). An inclusive grouping of proteobacteria is poorly supported (18%), but monophyletic epsilon- (99%) and alpha-proteobacterial (71%) clades are recovered, while the beta- and gamma-Proteobacteria form a single modestly supported (62%) clade within which many gamma-Proteobacteria constitute a solid (100%) sub-clade. By contrast, unconvincing JK values are recovered for several nodes along the subtree that joins the beta- and remaining gamma-Proteobacteria. There are two major discrepancies between our 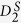 tree and the MRP reference: the Spirochaetales no longer form a monophyletic clade (*Treponema pallidum* is attracted to the problematic^8^ *Aquifex* and *Thermotoga* genomes), while our Crenarchaeota becomes non-exclusive (30%) with the inclusion of *Nanoarchaeum*. Overall, basal nodes exhibit lower JK support than do the more-terminal and leaf nodes, as is often the case for MSA-based trees of similar phyletic depth. As has been seen before^41^, most of the topological differences between the AF tree (Fig. 6) and the reference supertree arise from how bipartitions are resolved on short branches within major groups, not from disagreement about the membership of these groups.

#### 27 Escherichia coli and Shigella genomes

**Figure 7:**
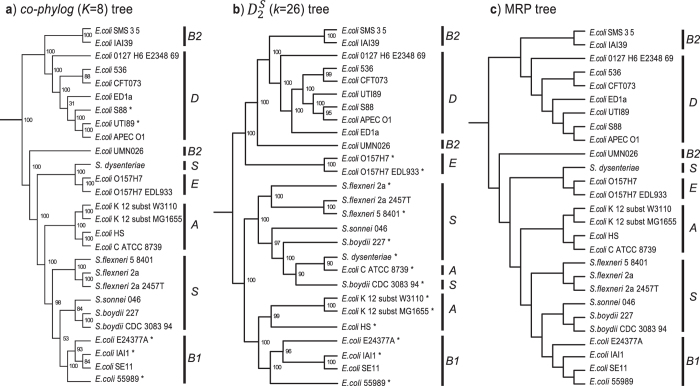
Phylogenetic trees of 27 *E. coli* and *Shigella* species. (**a**) Tree generated using *co-phylog* at *K* = 8, supported by JK values. (**b**) Tree generated using 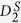 at *k* = 26, supported by JK values. (**c**) MRP tree constructed from 5282 Bayesian protein trees. Taxa labeled with an asterisk in each AF tree (**a,b**) are positioned differently in comparison to the reference (**c**). ECOR groups and *Shigella* (S) are indicated.

Figure 7 shows the phylogenetic tree of 27 *E. coli* and *Shigella* genomes generated using *co-phylog* (*K* = 8; Fig. 7a) or 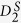 (*k* = 26; Fig. 7b), as well as the reference MRP supertree that summarises 5282 single-copy protein trees^3^ (Fig. 7c). These 27 taxa have been assigned to six distinct groups (the *E. coli* reference, or ECOR, strains)^3^,^42^ based on allelic diversity of 11 genes^43^. Each ECOR group is monophyletic, except B2 in both AF trees and A in the 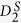 tree; the relationships among the ECOR groups in the *co-phylog* and MRP trees are identical. The taxa labeled with an asterisk in each AF tree (Fig. 7a,b) are positioned differently in comparison to the reference. In comparison to the reference (Fig. 7c), the same relationship among the six phyletic groups is recovered in the tree generated using *co-phylog* (Fig. 7a) at strong JK support (≥84% in all but two nodes), with minor topological difference within Groups B1 and D (*RF* = 0.083): *E. coli* 55989 (instead of E24377A) is placed as basal lineage in Group B1, while *E. coli* UTI89 (instead of *E. coli* S88) is placed as sister to *E. coli* APEC O1 in Group D. On the other hand, the tree generated using 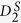 (Fig. 7b; all nodes JK ≥ 90%) is less congruent with the reference (*RF* = 0.45); all *Shigella* isolates (noted as Group S) form a strongly supported clade (JK = 100%) with *E. coli* ATCC 8739, while in the reference tree *Shigella dysenteriae* is placed externally to the other *Shigella* isolates, as sister to Group E, the pathogenic *E. coli* O157:H7 isolates. As shown in Fig. 7b, groups A and B1 are sister groups, supporting previous studies^42^,^44^ (Fig. 7b) and group E is placed within a strongly supported clade (JK 100%) together with isolates of Group B2 and D, and not with Groups A, B1 and S as shown in the reference (Fig. 7c).

#### Eight *Yersinia* genomes

**Figure 8:**
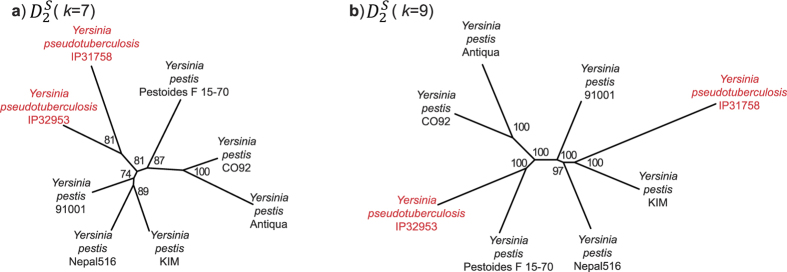
Phylogenetic trees of 8 *Yersinia* genomes. (**a**) Tree generated using 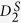 at *k* = 7, supported by JK values. (**b**) Tree generated using 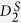 at *k* = 9, supported by JK values.

Figure 8 shows the phylogenetic trees based on 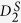 at *k* = 7 (Fig. 8a) and *k* = 9 (Fig. 8b). These topologies are well-supported, with all nodes showing JK ≥ 74% and ≥97% respectively. Topologically the *k* = 7 tree is more congruent with the reference, which was based on inversion events^5^, than is the *k* = 9 tree. The two *Y. pseudotuberculosis* isolates are sisters (81%) in the *k* = 7 tree, as in the reference^5^. At *k* = 9, on the other hand, *Y. pseudotuberculosis* IP31758 groups solidly (100%) with *Y. pestis* KIM relative to the others. In the whole-genome alignment (generated using progressiveMauve in the previous study^5^; Supplementary Figure S7) the region of the *Y. pseudotuberculosis* IP31758 genome between about 1.3–3.3 Mbp shares more-similar configuration of locally collinear blocks^5^ with the genome of *Y. pestis* KIM than with the genome of *Y. pseudotuberculosis* IP32953 (e.g. the corresponding region is largely in reverse complement). The two *Y. pseudotuberculosis* genomes are the most dissimilar in this set, sharing only 40.5% of all 12-mers present in these two genomes, whereas *Y. pseudotuberculosis* IP31758 and *Y. pestis* Pestoides F 15–70 are the most similar (72.4% of shared 12-mers; Supplementary Table S1). Thus depending on the value of *k*, 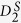 can draw out phylogenetic signal that supports either the species classification (Fig. 8a) or the major rearrangement events apparent in the whole-genome alignment (Fig. 8b).

### Computational efficiency and scalability

Table 1 shows the computation time (*t*) taken by each AF method to generate pairwise distance matrices for each of the three empirical datasets described above, using a single CPU and only one thread for the multi-threaded methods (e.g. 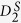 and *spaced*). For each method, *t* increases with *N*. The approach using *ffp* is the fastest, requiring *t* = 0.11, 0.65 and 2.22 minutes at *N* = 8, 27 and 143, while the *spaced* and *acs* methods are the most computationally intensive among these nine methods. The requirement for memory varies over two orders of magnitude, from less than 500 Mb for *kmacs* to more than 60 Gb for *acs* and 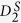 at *N* = 143.

## Discussion

In this study we demonstrate that AF phylogenetic approaches can be used to quickly and accurately infer phylogenomic relationships of microbes using whole-genome data. We also introduce for the first time a method, based on the jackknife, to provide node-support values in phylogenetic trees constructed using AF approaches.

We examined two types of AF methods in this study. In general, the methods based on word count, particularly *co-phylog* and 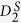, outperformed match-length methods. All these methods performed well on highly similar sequence data, but the methods based on word count (except *spaced*) are more robust as the input data become more divergent. All these AF methods proved robust against LGT at frequencies below *l* = 250 (at which every gene is likely to have affected by LGT at least once)^38^, antiquity of LGT and genome-scale rearrangement, extending our earlier results based on analysis of gene-scale sequence data^17^. Parameter values in this study were optimised for these data, based on our results and (for other AF methods) the original authors’ recommendations. These parameters are most sensitive to sequence length and genome divergence. AF methods have the advantage of being computationally fast, so we recommend that users compare a range of parameters on this basis.

The jackknife approach was simple to apply without prior sequence alignment. Here we deleted 40% of the data because 40% was previously shown to provide a reasonable balance between the generation of useful replicates without totally losing phylogenetic signal in sequence data^45^,^46^. The resulting JK support values appear to be biologically meaningful, as recognised taxa were often strongly supported (see also Supplementary Information and Supplementary Table S2). Particularly in the 143-genome dataset, JK support tended to decrease with increasing sequence divergence within-group, as is also seen with bootstrap support and Bayesian posterior probability in MSA-based studies. Problematic clades tend to be more weakly supported; for example, the beta- and gamma-Proteobacteria (JK 62% in Fig. 6) were the most-frequent LGT partners in the MSA-based study of this dataset^8^. Similarly, the appearance of *Pseudomonas* genomes within a clade of beta-Proteobacteria (JK 62%) can be explained by more than 150 LGT events^8^. Our AF tree also supports the hypothesis that Archaea is monophyletic (41%) and distinct from Bacteria^47^ (JK support 90% excluding the single representative (*Rhodopirellula*) in this dataset of the problematic Planctomycetes)^48^.

Likewise, AF trees based on the 27-genome *E. coli* and *Shigella* dataset recover the ECOR reference groups^3^. We observed two distinct trees: one congruent with the MRP supertree, and another that supports a monophyletic *Shigella* and the sister-group relationship between ECOR groups A and B1 as previously expected^44^ but not recovered in the supertree. As described above, relationships among the *Yersinia* strains cannot be resolved using a standard sequence-based approach: the 16 S ribosomal RNA sequence is identical for seven of these isolates, while the eighth (*Y. pseudotuberculosis* IP31758) differs in only five positions. All our AF-based methods (Fig. 8 and Supplementary Information) recovered only two types of topology, one of which reflects the extent of genome rearrangement among these strains. Given the intricate evolution of these taxa and the fact that we cannot recreate history the true phylogeny in these instances remains an open question.

Our use of an MRP supertree as reference is not entirely unproblematic in this context. A supertree summarises the well-supported topological features in a set of input trees; in these two cases^3^,^8^ and many others, each input tree arises via a workflow of orthogroup identification, MSA and Bayesian phylogenetic inference. Even in microbial genomes, where protein-coding genes tend to be tightly packed, many regions are excluded from contributing to the reference topology including non-protein-coding, intergenic, rearranged, mis-assembled, unalignable or low-complexity regions, paralogs, pseudogenes and repetitive elements. By contrast, AF approaches make use of the entire genome sequence and are less dependent on pre-defined evolutionary models; assumptions implicit in these models are known to be highly simplified and may be unrealistic^9^.

The computational complexity of all the AF methods can be found in previous studies^25^,^26^,^27^,^30^,^31^,^34^,^36^ and ranges from *O*(*n*) for *ffp*, to *O*(*k* × *n* × *z*) for *kmacs*, with *n* the number of sequences, *k* the number of mismatches and *z* the average number of matches between two sequences. An advantage that AF methods have over the standard MSA-based approaches is their scalability^15^,^17^. Among the AF methods we tested, methods based on word counts can be orders of magnitude faster than those based on match lengths, but tend to be more memory-intensive and more sensitive to parameter settings. It has been proposed^49^ that filtering out non-informative *k*-mers can be a useful approach to reducing memory requirements, and one can imagine a systematic, adaptive approach to parameterisation of *k* based on the nature of the sequence data (e.g. extent of divergence, presence of repetitive elements) as learned from simulated sequences. Biologically motivated approaches could likewise be explored, e.g. the adaptive use or weighting of *k*-mer patterns common in genomes (as learned from a comprehensive genome *k*-mer database) and/or relevant to biological processes or molecular function.

In cases where assessment of aligned positions across conserved regions is necessary, e.g. to infer structural features of proteins, MSA remains indispensable. However, these and other results show that AF approaches present exciting alternatives in phylogenetic inference for large sets of microbial-sized genomes at different phyletic breadth, even in the presence of genomic rearrangement and LGT. Indeed it might be asked whether AF approaches might be modified to detect genomic regions of lateral origin; we intend to address this in the very near future.

## Methods

### Simulated genome data

For all programs mentioned below, default settings were used unless otherwise specified. We simulated sets of genomes using two different programs depending on their functionality: Evolsimulator^38^ to simulate scenarios relevant to lateral genetic transfer (LGT), and ALF^50^ to simulate genome rearrangement. Evolsimulator allows the user to specify rates independently for speciation, extinction, gene content, extent of gene loss and/or duplication, nucleotide substitution and LGT. Moreover, LGT events can be allowed to succeed automatically, or can be evaluated under different criteria (e.g. controlled by gene complement, divergence, G + C similarity and/or host habitat); the receptivity of each genome to LGT can also be specified. We used the Generalised Time-Reversible (GTR)^51^ substitution model (rate parameters *a* = 0.987, *b* = 0.11, *c* = 0.218, *d* = 0.243, *e* = 0.395) in all simulations. We detail our simulation strategy for each evolutionary scenario below.

### Sequence divergence and lateral genetic transfer

Each set of genomes was simulated under a birth-and-death model at speciation rate = extinction rate = 0.5. The number of genomes in each set was allowed to vary from 25 to 35, with each containing 2000–3000 genes of length 240–1500 nucleotides. LGT receptivity was at set at minimum 0.2, mean 0.5 and maximum 0.8, mutation rate *m* = 0.4–0.6 and number of generations *i* = 5000. Sequence change was simulated under a discrete approximation of the gamma distribution (shape parameter 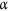 = 1.0, 8 categories). To study the effect of LGT we simulated LGT between randomly selected genomes, varying the mean number of LGT events attempted per iteration *l* = 5, 25, 125, 250 or 500 (each scenario in 50 replicates). We also simulated datasets in which genomes were not selected at random (but instead were restricted by number of generations from their common ancestor) by varying the divergence factor *d*: 200, 1000, 3000 or 5000 (with *l* fixed at 5, *m* 0.4–0.6); larger *d* allows more-dissimilar sequences to be transferred. To assess the effect of genome divergence we set *l* = 5 and simulated datasets at three rates of mutation *m*: 0.01–0.2, 0.4–0.6 and 0.8–0.99. All other parameters in this simulation follow Beiko *et al*.^52^.

### Genome rearrangement

We simulated different extents of rearrangement (random events of inverted translocation) within genome sequence sets using ALF, setting the rate *r* = 0.00, 0.01, 0.10 or 1.00 (each in 50 replicates). These values correspond to zero and approximately 0.2%, 2.0% and 20% of the length of each genome undergoing rearrangement. Other settings were number of genomes in each set *N* = 30 (each with 2500 genes of length 240–2000 nt), speciation rate = 0.5, extinction rate = 0.1, and mutation rate = 0.5. We allowed up to 20 genes to be involved in each inverted translocation event, following a uniform distribution: i.e. there was a 5% chance for one gene to be involved, a 5% chance for two genes to be involved, and so on.

### Empirical genome data

All genome datasets were obtained from NCBI (ncbi.nlm.nih.gov) and are based on published studies. The dataset of 143 diverse bacterial and archaeal genomes is a subset of the 144 genomes used in a previous study^8^ to detect highways of gene sharing; two genomes in that study, *Agrobacterium tumefaciens* Cereon and C58, have since been merged into a single record in the NCBI database (as *Agrobacterium fabrum* str. C58). The 27 *Escherichia coli* and *Shigella* genomes, and the eight *Yersinia* genomes, were taken respectively from Skippington and Ragan^3^ and Darling *et al*.^5^.

### Phylogenetic trees

For each genome set, we used nine AF methods to compute a phylogenetic tree. These methods all into two groups: word-count (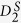, *cvt*, *ffp*, *co-phylog*, *spaced*) and match-length methods (*gram*, *acs*, *kr*, *kmacs*) (see Supplementary information). Six methods require a key parameter value to be set: *k*-mer length *k* for 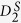, *ffp* and *cvt*; half-context length *K* for *co-phylog*; number of patterns *n* for *spaced*; and number of mismatches *mm* for *kmacs*. We tested wide range of values for these parameters (Supplementary Information) but for reasons of space report only the results obtained using the optimal parameter value, defined as that yielding the topology most congruent to the reference tree (see text). The distance matrix generated by each AF method was input into the neighbour-joining algorithm (*neighbor* in PHYLIP v3.69: evolution.genetics.washington.edu/phylip) to generate the corresponding tree.

### Jackknifing technique

For the jackknifing procedure we adapted the approach used by Shi *et al*.^46^ and used a jackknife rate of 40% as suggested by the authors and our tests on different jackknife rates (see Supplementary information and Supplementary Figure S8). From each genome set we generated 100 pseudo-replicates: for each pseudo-replicate a randomly selected 100-nt fragment was deleted from each genome, and this was iterated to a total of *N* times where *N* = (genome length × 0.4)/100, resulting in the deletion of (a different) 40% of each genome in each set. Then we generated a phylogenetic tree for each replicate using an AF method, and calculated node support by comparing the pseudo-replicate trees and the test tree.

### Assessment of accuracy

To assess the accuracy of each AF method we computed the Robinson-Foulds distance between a tree computed using that method (the “test tree”) and the corresponding reference tree, using *treedist* in PHYLIP v3.69. This distance represents the number of bipartitions that are present in only one of the two trees. To facilitate comparison of our results sequence sets (hence trees) of different sizes *N*, following Kupczok *et al*.^53^ we normalise this distance according to the maximum possible distance between two unrooted trees, 2(*N* − 3). We denote this normalised Robinson-Foulds distance as *RF*, with a value from 0 to 1 that can be interpreted as the proportion of false or missing bipartitions in the test-tree topology compared to the reference topology. When *RF* = 0 the test and reference topologies are identical, implying high accuracy for the method. Conversely, at *RF* = 1 no bipartition in the reference is recovered. A pair of randomly generated tree topologies of *N* taxa has a Robinson-Foulds distance that approaches the denominator for normalisation, 2(*N* − 3)^54^, i.e. when *RF* = 1. For the simulated data we used as reference the known tree (according to which the sequences were simulated) provided by Evolsimulator or ALF. For the *E. coli* - *Shigella* and 143-genome empirical datasets we used as reference the MRP supertrees from Skippington and Ragan^3^ and Beiko *et al*.^8^ respectively; in these cases *RF* does not inform directly on accuracy (as the true tree is unknown), but instead reflects the extent to which the AF method recovers the published topology. For the *Yersinia* dataset we used the consensus phylogenetic network^5^ as reference.

### Computational scalability and runtime

Assessment of computational scalability was carried out using a high-performance distributed-memory computing cluster based on Intel Xeon ‘Haswell’ Cores 3.1 GHz. Comparative runtime analysis of alignment-free methods was done on Intel Xeon ‘Haswell’ Cores (E5-2667 v3) @ 3.1 GHz/boost 3.5 GHz (using a single processor and one thread).

## Additional Information

**How to cite this article**: Bernard, G. *et al*. Alignment-free microbial phylogenomics under scenarios of sequence divergence, genome rearrangement and lateral genetic transfer. *Sci. Rep.* **6**, 28970; doi: 10.1038/srep28970 (2016).

## Supplementary Material

Supplementary Information

## Figures and Tables

**Figure 1:**
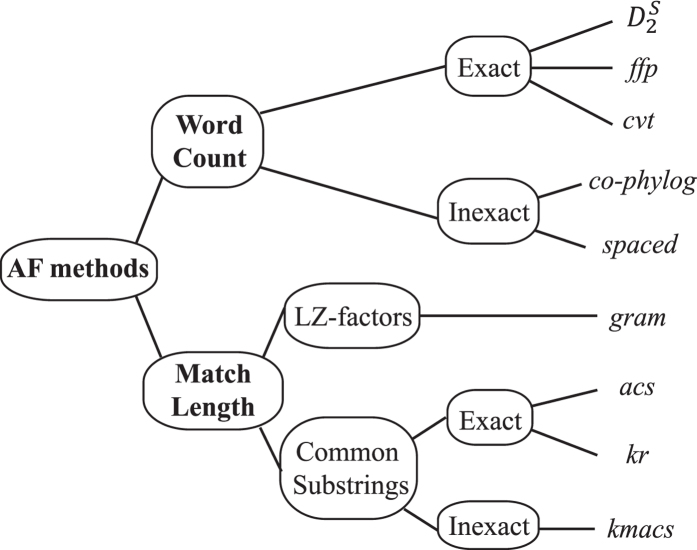
Alignment-free methods classification. Classification of alignment-free methods, modified following Haubold^15^. LZ: Lempel-Ziv.

**Table 1:** Computation time required by the nine AF methods.

AF method	*N* = 8	*N* = 27	*N* = 143
*co-phylog*	0.37	3.47	82.80
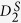	2.21	9.06	20.35
*cvt*	4.80	37.15	179.76
*ffp*	0.11	0.65	2.22
*spaced*	12.67	154.42	12713.54
*acs*	18.67	110.75	1197.71
*gram*	55.34	212.33	723.87
*kmacs*	13.82	261.55	594.44
*kr*	0.93	5.16	28.34

The computation time, *t* (in minutes) for all the methods across three empirical datasets (*N* = 8, 27 and 143). Mean genome lengths (with standard deviation) are 4.634 ± 0.080, 4.906 ± 0.294 and 3.011 ± 1.802 Mb for these datasets respectively. We set *k* = 16 for 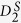, *cvt* and *ffp*, *K* = 7 for *co-phylog, n* = 60 for *spaced* and *mm* = 12 for *kmacs.*
