# Supplementary material for: Alignment-free microbial phylogenomics under scenarios of sequence divergence, genome rearrangement and lateral genetic transfer
Source: Sci Rep. 2016 Jul 1;6:28970. doi: 10.1038/srep28970 (PMC4929450; doi:10.1038/srep28970)
Supplement: Supplementary Information [file srep28970-s1.pdf]

Supplementary Information

Alignment-free microbial phylogenomics under scenarios of sequence divergence, genome rearrangement and lateral genetic transfer

Guillaume Bernard[1], Cheong Xin Chan[1] & Mark A. Ragan[1,*]

[1]Institute for Molecular Bioscience, and ARC Centre of Excellence in Bioinformatics, The University of Queensland, Brisbane, QLD 4072, Australia, *Corresponding author.

Alignment-free methods used in this study

Word-count methods

D_2^S **statistic.** D_2^S generate a score for each possible pair of sequences within a set [1] based on *k*-mer count. These scores are transformed *via* logarithmic representation of the geometric mean to generate a distance [2]. Generation of the distance matrix using D_2^S is implemented in a JAVA program, jD2Stat [2], which is freely available at bioinformatics.org.au/tools/jD2Stat/.

Feature frequency profiles. The *ffp* pipeline [3-5] builds the *k*-mer frequency profile for each sequence, and then uses the Jensen-Shannon divergence [4] to compare their profiles and generate a distance between the sequences.

Composition vector. The composition vector method *cvt* shares the same principle as *ffp*, but here the *k*-mer frequencies are divided by the frequencies expected by chance alone [6].

Co-phylog. This method counts the proportion of shared words that differ in the middle position as a distance between two sequences, i.e. a mutation point surrounded by a context of certain length present in both sequences (with *K*, the half-context length at each side of the mutation point) [7].

Spaced-word frequencies. *Spaced* uses sets of match and mismatch position patterns to compare the spaced-word frequencies between two sequences. The patterns can vary in length and number *n* [8,9].

Match-lengths methods

Grammar-based method. The method *gram* uses a set of rules to decompose a string into substrings, e.g. CCCTTTAA decomposed as C3T3A2. The idea is that closely related sequences are more compressible than divergent sequences [10]. Different compression schemes can be used, but Lempel-Ziv factorisation (used by the gzip compression) is the most common [11].

Average Common Substring. The Average Common Substring (*acs*) method [12] uses the concept of matching statistics [13]. Instead of decomposing the concatenation of two sequences, this method searches for the longest match in *sequence A* starting at every position in *sequence B*. Unlike the Lempel-Ziv factorisation method, here the longest matches can overlap.

Shortest unique substring. Instead of looking for the longest matches between two sequences, *kr* looks for the longest common substrings extended by one, known as the longest substrings between two mutations, i.e. the SHortest Unique subSTRING, *shustring* [14].

***k*-Mismatch Average Common Substring (*kmacs*).** This method is a variant of the *acs* method, using the longest common substring with *k*-mismatches (the number of mismatches is noted *mm* in our study) [9,15].

Optimisation of parameter settings

Six of the nine AF methods used in this study require the specification of a key parameter that would influence the estimated distance among a set of sequences, thus the resulting trees. D_2^S, *ffp* and *cvt* require a value to be set for *k* (i.e. *k*-mer length), *co-phylog* requires a half-context length *K*, *spaced* a number of patterns *n*, and *kmacs* a number of mismatches *mm*. To assess the optimal parameter setting corresponding to each of these methods in inferring phylogenies across the simulated data, we ran the program across a range of values and inferred a phylogenetic tree. We used *k* = [14-26] for D_2^S, *ffp* and *cvt*, *K* = 6, 7, 8 and 9 for *co-phylog*, and based on authors' recommendations [15], *n* = 60, 70, 80, 90 and 100 for *spaced*, and *mm* = [12-20] for *kmacs.*

We consider, in each scenario, the parameter that yielded the minimum average *RF* (i.e. the tree topology that is the most congruent to reference) as optimal. The other three methods, *gram*, *kr* and *acs* were run using the default parameters.

Analysis of genome divergence. Supplementary Fig. S1 shows the mean *RF* observed using the six AF methods across different parameter settings and the mutation rate *m*. For D_2^S (Supplementary Fig. S1a), *ffp* (Supplementary Fig. S1b) and *cvt* (Supplementary Fig. S1c), the optimal *k*-mer length slightly increases proportionately as *m* (e.g. for *cvt*, the optimal *k* = 17, 20 and 21 at *m* = 0.1, 0.5 and 0.9). Minimum *RF* values observed using optimal *k* in these methods do not differ by much and are relatively small, i.e. <0.05 in most cases. For *co-phylog* (Supplementary Fig. S1d) the best *K* is either 6 or 7 depending on *m*. For *spaced* (Supplementary Fig. S1e), varying *n* yielded very similar *RF* values (e.g. *RF* = 0.5 across all *n* examined at *m* = 0.9). Finally, for *kmacs* (Supplementary Fig. S1f), the optimal *mm* increases as *m* increases, e.g. optimal *mm* = 12, 19 and 20 at *m* = 0.1, 0.5 and 0.9; the difference in *RF* values across *mm* examined is relatively small, i.e. <0.025 across incremental *mm* in each case.

Analysis of lateral genetic transfer. Supplementary Fig. S2 shows the mean *RF* obtained using the six AF methods across different parameter settings and the LGT per iteration rate *l*. For D_2^S (Supplementary Fig. S2a) and *cvt* (Supplementary Fig. S2c) the optimal *k*-mer lengths decrease as *l* increases (e.g. optimal *k* = 17, 15 and 14 at *l* = 0, 125, 500 for D_2^S). Minimum *RF* values observed using optimal *k* in these methods do not differ by much and are relatively small, i.e. < 0.05 across incremental *k*. For *ffp*, *co-phylog* and *kmacs* (Supplementary Figs S2b, S2d and S2f) the optimal parameter is always the same at different *l* values, i.e. optimal setting *k* = 20 for *ffp*, *K* = 6 for *co-phylog* and *mm* = 20 for *kmacs*. Finally, for *spaced* (Supplementary Fig. S2e), the use of different *n* settings yielded similar *RF* values, e.g. optimal *n* is the same across all tested values (60, 70, 80, 90 and 100) at *l* = 0, 25 and 250.

Supplementary Fig. S3 shows the mean *RF* obtained using the six AF methods across different parameter settings and the divergence factor *d*. For all the methods, the observed variation of *RF* is very little between the best parameters, e.g. for *cvt*, *RF* between 0.032-0.037 and 0.028–0.033 at *k* = 20 and 22 respectively (Supplementary Fig. S3c). Similarly to

what we observed in Supplementary Figs S1e and S2e, varying the setting of n in *spaced* yielded almost identical *RF*.

Analysis of genome rearrangements. Supplementary Fig. S4 shows the mean *RF* obtained using five of the six AF methods across different parameter settings and the rearrangement rate r. Owing to the highly consistent results observed in *spaced* using different values of n (as described above), we did not assess the setting of n in *spaced* in this case. In all methods, the observed variation of *RF* is very little between the best parameters, e.g. for *cvt*, *RF* ranges between 0.068 (r = 1.00) and 0.070 (r = 0.10) at k = 14 (Supplementary Fig. S4c).

To visualise the extent of inverted translocation across these datasets, we computed the MSA of these datasets using progressiveMauve [16]. Supplementary Fig. S5 shows an example of part of the whole genome alignments at each r. The number of locally collinear blocks [17] in Mauve alignments increases proportionately with r, illustrating the complexity in multiply aligning these genomes, particularly when $r \geq 0.1$.

Analysis of genome divergence in empirical data

To estimate the divergence among the 143 genomes, we first generated a phylogenetic tree using the D_2 method [18] at k = 26 (Supplementary Fig. S6). Because D_2 is a simple dissimilarity measure, the branch lengths of this tree indicate divergence (although not directly interpretable) of the species. The tree inferred by D_2 (Supplementary Fig. S6) shows that Archaea are separated (with long branch lengths within clade) from the Bacteria. The internal branch lengths are relatively shorter than the external branches, suggesting that multiple speciation events (i.e. an adaptive radiation process) occurred in a short timeframe after the establishment of corresponding niches (e.g. bacterial groupings).

We also compute the percentage of shared k-mers P_k for each genome pair across all empirical dataset (Supplementary Table S1), here with k =12. We calculate this percentage P_k as

$$P_k = \frac{S \times 100}{T}$$

where S is the sum of occurrences of shared k-mers in both genomes, and T is the sum of all possible k-mers in both genomes (i.e. $g - k + 1$, with g = genome length); a small value of P_k indicates high divergence among the genomes. If this k is too small all the genomes tend to share almost all k-mers, whereas if k is too large too few k-mers are shared, particularly on the 143-genome set. At $P_{k=12}$ we observe an appropriate range of similarities across the different datasets. The 143-genome set is the most divergent ($P_{k=12}$ = 15.94%), followed by *Yersinia* ($P_{k=12}$ = 61.39%) and *E.coli-Shigella* ($P_{k=12}$ = 64.06%).

Selection of jackknife rate for pseudo-replicates generation

Previous studies suggested that a jackknife "rate" (proportion) ρ = 37.5-40% is optimal for the jackknife technique[19,20], but this was based on aligned sequences or gene-order data. To determine which ρ was optimal for our jackknife (JK) analysis we used a range of 20-80% cut-off to generate 100 pseudo-replicates for each empirical dataset. We followed the same technique described in the main Methods to generate JK support values at each rate; the results are shown in Supplementary Fig. S8. The JK values decrease as ρ increases across the three empirical datasets, and across two different sizes of k for the *Yersinia* genomes. With a ρ below 40% we observed for all trees a mean JK value above 90%, with an almost perfect value of 100% for the *E. coli/Shigella* and *Yersinia* (at k=9) datasets. At ρ higher than 50-% we observed large variation for the JK values across all empirical datasets. At ρ = 40-50% we observed different levels of variation for the JK values based on the datasets: the JK values are above 90% for the *E. coli/Shigella* and *Yersinia* (at k=9) datasets, and above 80% for the 143-genome and *Yersinia* datasets (with a larger distribution for the 143-genome). Our finding suggests that ρ between 30-60% would give the best dynamic range of support values across different datasets; for that reason we decided to used 40% for our analysis.

Assessment of jackknife pseudo-replicates in different AF methods

To assess the robustness of jackknife support in each of the AF methods, we independently accessed topological difference, measured as *RF* (normalised Robinson-Foulds distance), between (a) a supertree that is summarised from 100 trees (independently generated from each JK pseudo-replicate of the same data), and (b) the tree generated using the AF method from the original (non-jackknifed) data (144 prokaryote genomes). Here we used three supertree methods: the maximum representation of parsimony (MRP) [21], the fast subtree Prune-and-Regraph (fast-SPR) [22] and the extended majority rule (exMR) [23]; the results are shown in Supplementary Table S2. We used the R package *phytools* [24] to generate the MRP supertree, SPRSupertrees (kiwi.cs.dal.ca/Software/SPRSupertrees) to generate the fast-SPR supertree, and the *consense* as implemented in PHYLIP v3.69 (evolution.genetics.washington.edu/phylip) to generate the exMR supertree.

We observed the lowest *RF* for D_2^S (*RF* = 0.04) and the highest for *kmacs* (*RF* = 0.45); there was little variation among the supertrees as generated using each of the three methods. Our results suggest that some AF methods (particularly D_2^S, *ffp* and *cvt*) are more robust to data truncation and therefore more appropriate for the calculation of jackknife support.

Supplementary References

1 Wan, L., Reinert, G., Sun, F. & Waterman, M. S. Alignment-free sequence comparison (II): theoretical power of comparison statistics. *J Comput Biol* **17**, 1467-1490, doi:10.1089/cmb.2010.0056 (2010).

2 Chan, C. X., Bernard, G., Poirion, O., Hogan, J. M. & Ragan, M. A. Inferring phylogenies of evolving sequences without multiple sequence alignment. *Sci Rep* **4**, 6504, doi:10.1038/srep06504 (2014).

3 Jun, S. R., Sims, G. E., Wu, G. A. & Kim, S. H. Whole-proteome phylogeny of prokaryotes by feature frequency profiles: An alignment-free method with optimal

feature resolution. *Proc Natl Acad Sci U S A* **107**, 133-138, doi:10.1073/pnas.0913033107 (2010).

4 Sims, G. E., Jun, S. R., Wu, G. A. & Kim, S. H. Alignment-free genome comparison with feature frequency profiles (FFP) and optimal resolutions. *Proc Natl Acad Sci U S A* **106**, 2677-2682, doi:10.1073/pnas.0813249106 (2009).

5 Sims, G. E. & Kim, S. H. Whole-genome phylogeny of *Escherichia coli/Shigella* group by feature frequency profiles (FFPs). *Proc Natl Acad Sci U S A* **108**, 8329-8334, doi:10.1073/pnas.1105168108 (2011).

6 Wang, H., Xu, Z., Gao, L. & Hao, B. A fungal phylogeny based on 82 complete genomes using the composition vector method. *BMC Evol Biol* **9**, 195, doi:10.1186/1471-2148-9-195 (2009).

7 Yi, H. & Jin, L. Co-phylog: an assembly-free phylogenomic approach for closely related organisms. *Nucleic Acids Res* **41**, e75, doi:10.1093/nar/gkt003 (2013).

8 Leimeister, C. A., Boden, M., Horwege, S., Lindner, S. & Morgenstern, B. Fast alignment-free sequence comparison using spaced-word frequencies. *Bioinformatics* **30**, 1991-1999, doi:10.1093/bioinformatics/btu177 (2014).

9 Leimeister, C. A. & Morgenstern, B. Kmacs: the *k*-mismatch average common substring approach to alignment-free sequence comparison. *Bioinformatics* **30**, 2000-2008, doi:10.1093/bioinformatics/btu331 (2014).

10 Russell, D. J., Way, S. F., Benson, A. K. & Sayood, K. A grammar-based distance metric enables fast and accurate clustering of large sets of 16S sequences. *BMC Bioinformatics* **11**, 601, doi:10.1186/1471-2105-11-601 (2010).

11 Haubold, B. Alignment-free phylogenetics and population genetics. *Brief Bioinform* **15**, 407-418, doi:10.1093/bib/bbt083 (2014).

12 Ulitsky, I., Burstein, D., Tuller, T. & Chor, B. The average common substring approach to phylogenomic reconstruction. *J Comput Biol* **13**, 336-350, doi:10.1089/cmb.2006.13.336 (2006).

13 Gusfield, D. *Algorithms on strings, trees and sequences: computer science and computational biology*. (Cambridge university press, 1997).

14 Haubold, B., Pierstorff, N., Moller, F. & Wiehe, T. Genome comparison without alignment using shortest unique substrings. *BMC Bioinformatics* **6**, 123, doi:10.1186/1471-2105-6-123 (2005).

15 Horwege, S. *et al. Spaced words* and *kmacs*: fast alignment-free sequence comparison based on inexact word matches. *Nucleic Acids Res* **42**, W7-11, doi:10.1093/nar/gku398 (2014).

16 Darling, A. E., Mau, B. & Perna, N. T. progressiveMauve: multiple genome alignment with gene gain, loss and rearrangement. *PLoS One* **5**, e11147, doi:10.1371/journal.pone.0011147 (2010).

17 Darling, A. C., Mau, B., Blattner, F. R. & Perna, N. T. Mauve: multiple alignment of conserved genomic sequence with rearrangements. *Genome Res* **14**, 1394-1403, doi:10.1101/gr.2289704 (2004).

18 Reinert, G., Chew, D., Sun, F. & Waterman, M. S. Alignment-free sequence comparison (I): statistics and power. *J Comput Biol* **16**, 1615-1634, doi:10.1089/cmb.2009.0198 (2009).

19 Lapointe, F. J., Kirsch, J. A. & Bleiweiss, R. Jackknifing of weighted trees: validation of phylogenies reconstructed from distance matrices. *Mol Phylogenet Evol* **3**, 256-267, doi:10.1006/mpev.1994.1028 (1994).

20 Shi, J., Zhang, Y., Luo, H. & Tang, J. Using jackknife to assess the quality of gene order phylogenies. *BMC Bioinformatics* **11**, 168, doi:10.1186/1471-2105-11-168 (2010).

21 Ragan, M. A. Phylogenetic inference based on matrix representation of trees. *Mol Phylogenet Evol* **1**, 53-58, doi:10.1016/1055-7903(92)90035-F (1992).

22 Whidden, C., Zeh, N. & Beiko, R. G. Supertrees Based on the Subtree Prune-and-Regraft Distance. *Syst Biol* **63**, 566-581, doi:10.1093/sysbio/syu023 (2014).

23 Margush, T. & McMorris, F. R. Consensus-trees. *Bulletin of Mathematical Biology* **43**, 239-244 (1981).

24 Revell, L. J. phytools: an R package for phylogenetic comparative biology (and other things). *Methods in Ecology and Evolution* **3**, 217-223, doi:10.1111/j.2041-210X.2011.00169.x (2012).

Supplementary Table S1. The mean $P_{k=12}$ value (and standard deviation) for each empirical dataset, with the minimum and maximum genome pairs.

Dataset	Average Genome length (Mb) ± SD	Mean ± SD	Minimum *Pk* (taxa)	Maximum *Pk* (taxa)
Yersinia genomes	4.634 ± 0.080	61.39 ± 8.00	40.52 (*Y .pseudotuberculosis* IP32953 and IP31758)	72.43 (*Y. pseudotuberculosis* IP32953 and *Y. pestis* pestoidF)
E.coli/Shigella genomes	4.906 ± 0.294	64.06 ± 10.58	37.15 (*E. coli* HS and ATCC_9739)	94.51 (*E. coli* S88 and APEC_01)
143 genomes	3.011 ± 1.802	15.94 ± 10.44	0.42 (*Wigglesworthia* and *Streptomyces coelicolor*)	99.45 (*Chlamydophila pneumoniae* TW 183 and CWL029)

Supplementary Table S2. *RF* between the tree generated using each AF method and each of the three supertrees generated using the corresponding AF method, summarised from 100 JK pseudo-replicates.

AF method	Supertree method		
	MRP	fast-SPR	ExMR
D_2^S (*k*=16)	0.04	0.04	0.04
ffp (*k*=16)	0.09	0.11	0.09
cvt (*k*=18)	0.07	0.16	0.14
gram	0.19	0.15	0.15
spaced (n=60)	0.24	0.24	0.24
co-phylog (*K*=8)	0.29	0.29	0.33
kr	0.37	0.44	0.41
kmacs (*mm*=12)	0.44	0.45	0.45
